# Subacromial impingement syndrome in a patient with hereditary multiple exostosis: a case report

**DOI:** 10.1186/2052-1847-5-20

**Published:** 2013-10-11

**Authors:** Nicholas D Clement, Julie M McBirnie, Daniel E Porter

**Affiliations:** 1 Department of Orthopaedics and Trauma Surgery, Royal Infirmary of Edinburgh, Little France, Edinburgh EH16 4SA, UK

**Keywords:** Exostosis, Shoulder, Impingement, Arthroscopy

## Abstract

**Background:**

Hereditary multiple exostosis (HME) is characterised by multiple osteochondromas that are distributed throughout the skeleton, invariably involving the shoulder girdle. Tumours within the subacromial space can cause secondary irritation of the rotator cuff and result in subacromial impingement syndrome.

**Case presentation:**

We describe a 19 year old female patient with HME who presented with subacromial impingement syndrome secondary to a benign exostosis originating from the spine of the scapular and projecting into the subacromial space.

**Conclusion:**

The unique aspects of this report was that the origin of the exostosis, which was not observed on early standard radiographs of the shoulder, and the use of arthroscopic excision of the exostosis. Hence we believe a low threshold for additional imaging, such as a magnetic resonance imaging, should be considered for patients with HME with subacromial impingement syndrome to ensure a symptomatic exostosis is not neglected. Arthroscopic excision of a benign subacromial exostosis is effective, offering a minimally invasive approach with relief of the patient’s symptoms.

## Background

Hereditary multiple exostoses (HME) is one of the most commonly inherited musculoskeletal conditions, with an incidence of one in 50,000 and demonstrates an autosomal dominant trait [1]. The exostoses are distributed throughout the skeleton, most commonly at the metaphysis of long bones [2]. Invariably the shoulder girdle is affected [3] and accounts for more than 14% of all palpable exostoses [4]. Whilst the majority of exostoses are asymptomatic complications can occur, which include pain, deformity and shortening of long bones, restricted joint motion, nerve or blood vessel compression [5]. Relative to all other exostoses there is an increased risk of surgical excision with shoulder exostoses, which is increased further still for exostoses of the scapula [4]. This is thought to be due to the subcutaneous nature of the scapula and interference of the scapulothoracic rhythm [4].

There are multiple case reports in the literature of exostoses arising around the shoulder, from the proximal humerus, scapula and ribs, in HME patients presenting with pain and dysfunction [6-9]. However, we found no reports of an exostosis arising from the spine of the scapular causing subacromial impingement. We present the case of a 19 year-old female patient with HME and subacromial impingement syndrome of the shoulder.

## Case presentation

A 19 year-old female nursing student presented to our clinic with a one-year history of persistent shoulder impingement symptoms despite conservative measures, describing a clicking and catching sensation associated with pain on rotation and abduction of her shoulder. She had a past medical history of HME. Her general practitioner had requested plain shoulder radiographs during this time which were reported as normal.

On examination she had symmetrical muscle bulk of her shoulder girdle with no wasting and a full range of movement of both shoulders. On the left side she had a painful arc sign. In addition there was a definite audible “clunk” at 45 degrees of external rotation of the shoulder in 90 degrees of abduction. There was also crepitus in the subacromial area throughout abduction. There was no suggestion of capsular tightness and she had a clinically intact rotator cuff with full strength.

**Figure 1:**
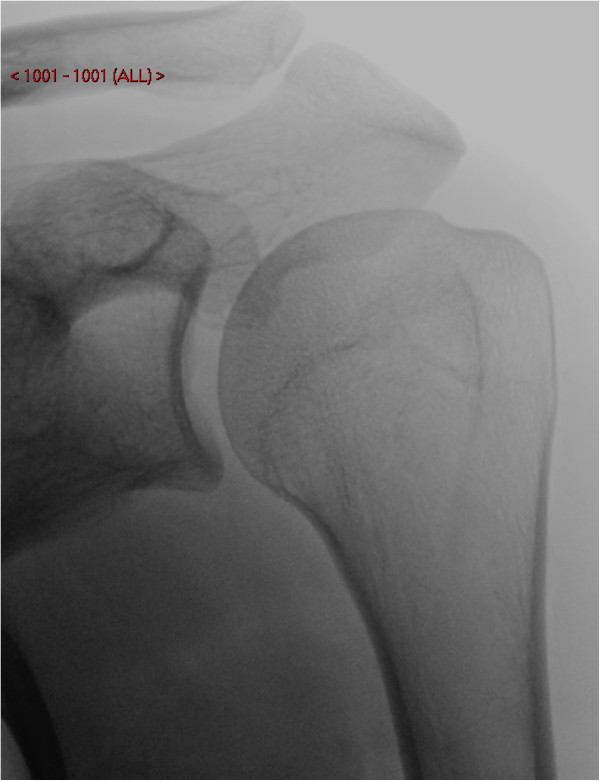
Anterior-posterior radiograph of the left shoulder.

**Figure 2:**
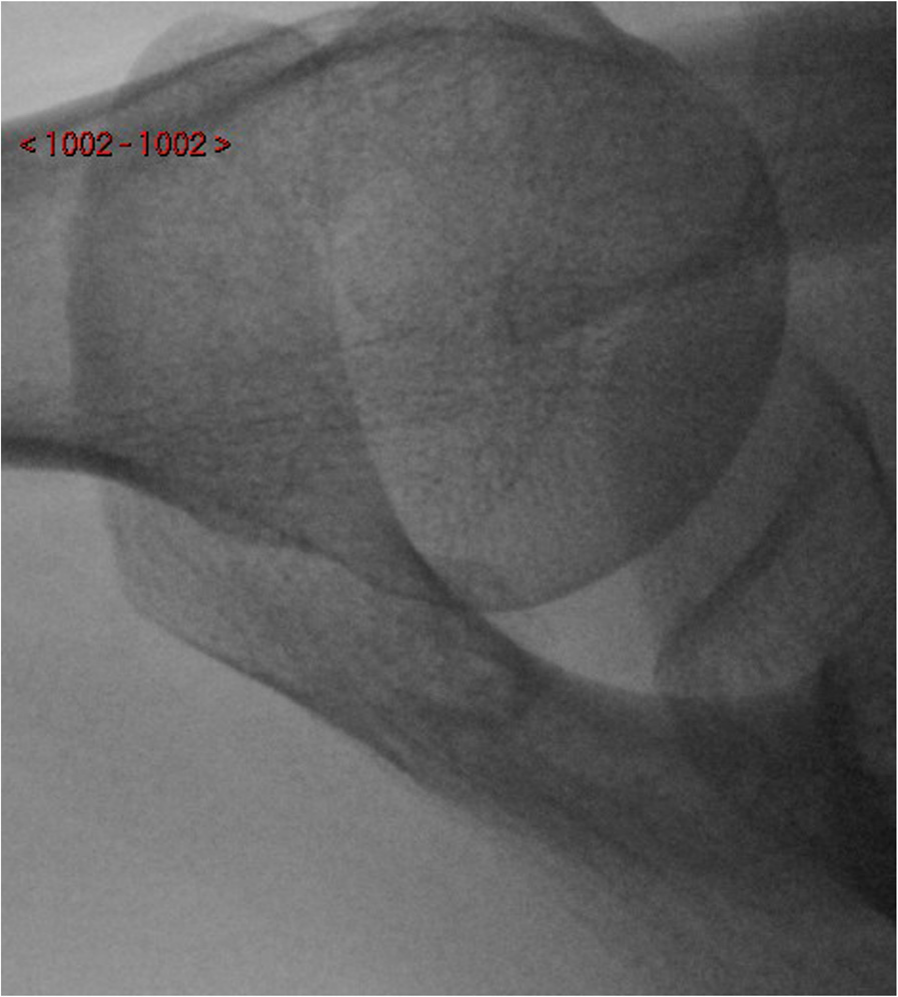
Axial radiograph of the left shoulder.

**Figure 3:**
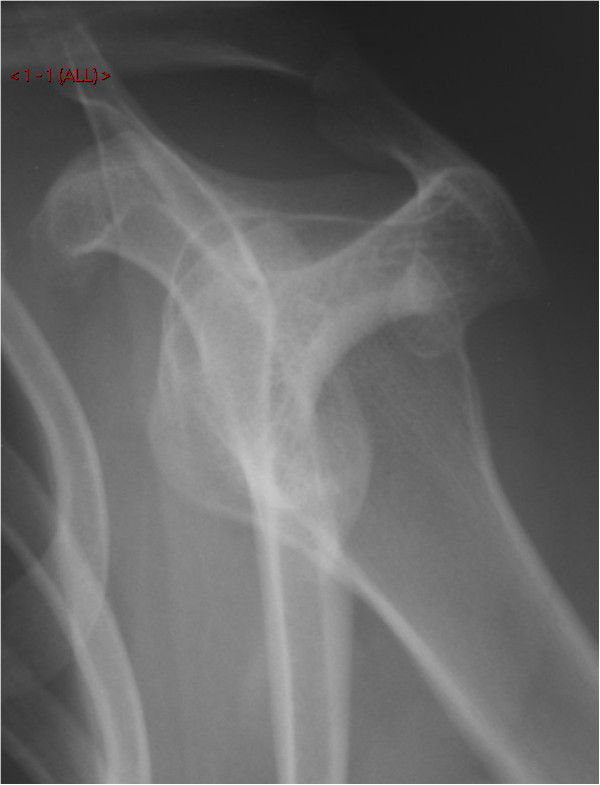
A lateral (scapular Y-view) radiograph of the left shoulder joint demonstrating the exostosis protruding into the subacromial space.

**Figure 4:**
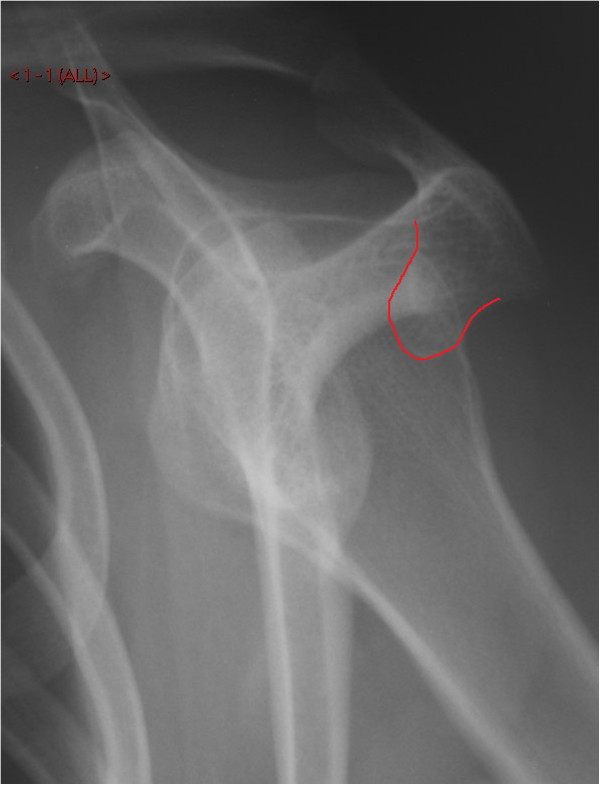
A lateral (scapular Y-view) radiograph of the shoulder joint demonstrating the exostosis protruding into the subacromial space which is outlined by the red line.

**Figure 5:**
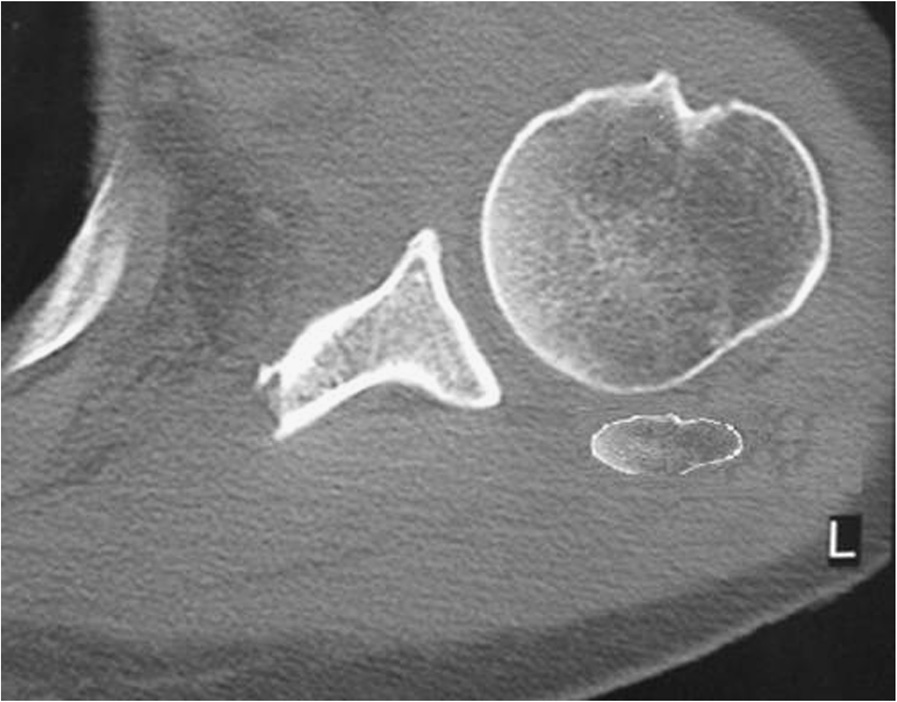
CT scan of the left shoulder demonstrating an axial cut through the shoulder at the level of the glenoid that illustrates the exostosis posterior to the humeral head in the subacromial space.

Plain anterior-posterior and axial radiographs of the shoulder were unremarkable upon initial inspection (Figures 1 and 2). However, due to clinical suspicion a lateral radiograph (scapular Y-view) of the shoulder was requested which demonstrated an exostosis at the posterior aspect of the scapula (Figures 3 and 4), which was confirmed to have a benign appearance on an magnetic resonance imaging (MRI) scan and computer tomography (CT) scan (Figure 5).

**Figure 6:**
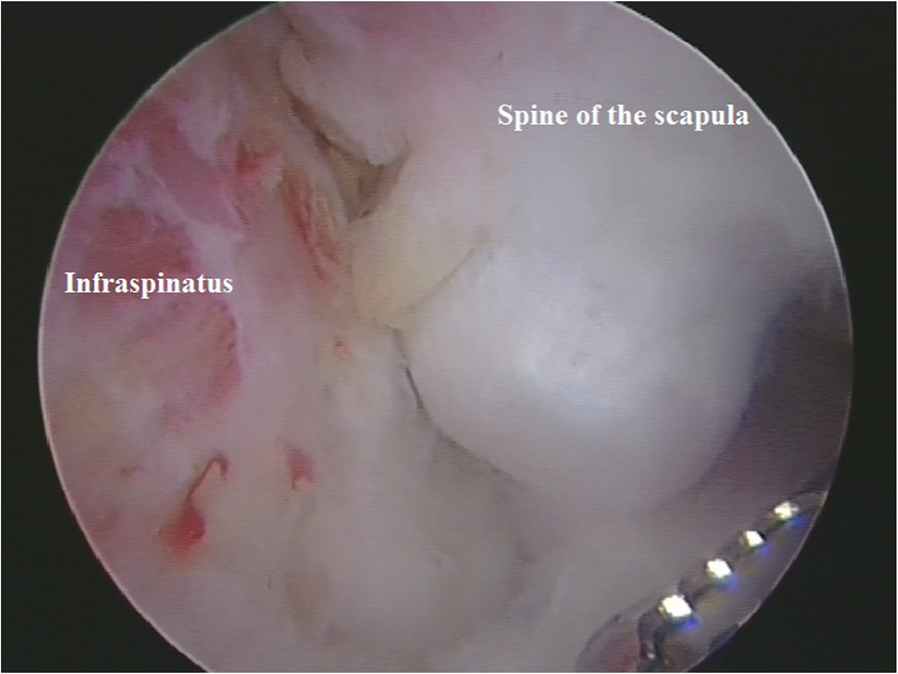
**Exostosis arising from the posterior-lateral aspect of the scapula spine projecting anteriorly into the subacromial space.** This arthroscopic view is from a lateral portal looking from distal to proximal.

**Figure 7:**
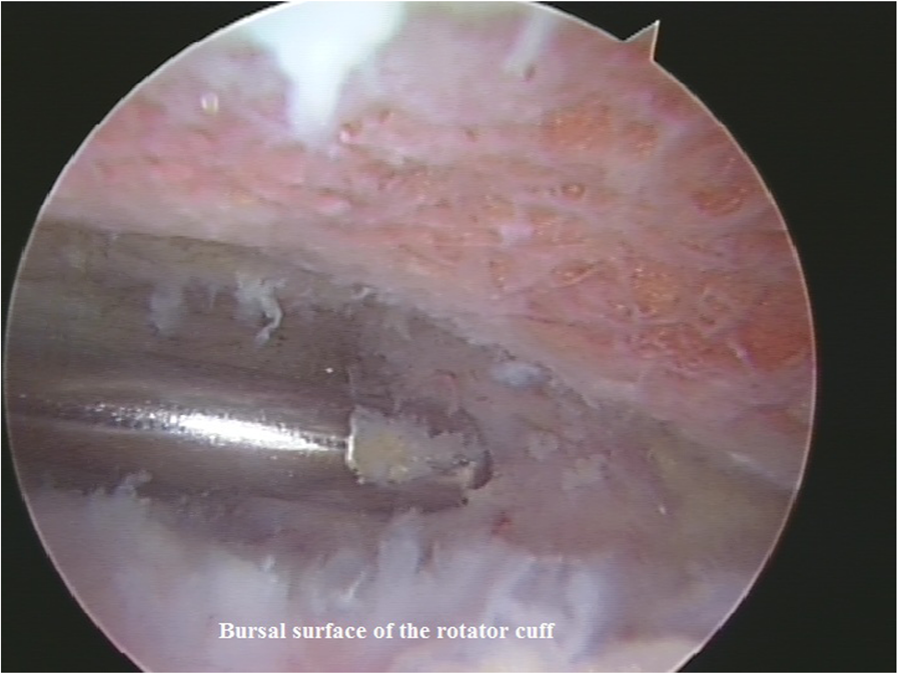
Excision of the exostosis using an arthroscopic burr.

The patient underwent arthroscopic shoulder surgery, which revealed an exostosis arising posteriorly from the lateral aspect of the scapula spine projecting anteriorly into the subacromial space (Figure 6). There was marked fibrosis within the subacromial space which was excised to aid visualization of the exostosis, we hypothesize that this was secondary to the persistent irritation and inflammation of the subacromial bursa. At 90 degrees of shoulder abduction and 45 degrees of external rotation the exostosis impinged upon the bursal surface of the rotator cuff. A biopsy was taken before excision which confirmed the benign appearance of the osteochondroma diagnosed on the MRI and CT scans. The exostosis was removed using an arthroscopic burr (Figure 7). Following the decompression she regained a full range of movement without impingement.

Immediate mobilization was commenced post-operatively under the supervision of a physiotherapist. At three months her symptoms had completely resolved with full pain free range of movement and shoulder strength. There has been no recurrence to date, 4 years after surgery.

## Discussion

The unique aspect of our case report is the origin of the exostosis, from the spine of the scapula, which was not obvious on standard anterior-posterior and axial radiographs of the shoulder. Only after a year of symptoms did the radiographic findings, using a scapular Y-view, reveal the exostosis that had caused her persistent symptoms. Furthermore, to our knowledge arthroscopic excision of an exostosis for subacromial impingement is original to our report, offering a minimally invasive approach with relief of the patient’s symptoms.

Craig [6] is the only other author to have reported a patient with HME suffering from subacromial impingement syndrome secondary to osteochondromas of the acromion and distal clavicle. However, similar to our case, their patient had persistent shoulder symptoms for one year before the exostoses, arising from the under surface of the acromion and the clavicle, were evident on the plain radiographs and were subsequently excised through an open approach giving symptomatic relief. Reichmister et al. [8] reported a further two cases of isolated osteochondromas of the distal clavicle, describing persistent symptoms of subacromial impingement, before being eventually diagnosed. The exostosis reported in our patient was not originally observed on early radiographs, and only with persistence of their symptoms was further imaging performed. This illustrates the importance in acquiring early additional imaging, such as a MRI scan, for patients with mechanical symptoms who have apparently normal initial radiographs. This additional imaging would seem more important in patients with HME who are more prone to suffer with symptomatic exostosis. However, soft-tissue tumours within the subacromial space can also present with impingement type symptoms in patients without HME, who may also benefit from an early MRI scan [10,11].

Artrhoscopic removal of the exostosis offers several advantages over open excision including less invasive surgery, an earlier recovery, and shorter hospital stay [12,13]. Arthroscopic excision of osteochondromas for the treatment of snapping scapula has previously been described [14,15]. The exostoses in patients with HME may be numerous, although many are asymptomatic and a cautious treatment approach is warranted with removal of symptomatic exostoses only [2,16]. Arthroscopic surgery, however, offers advantages to patients with HME and should be considered as a management option for the excision of symptomatic osteochondromas.

The lifetime risk of sarcoma in HME is thought to be approximately 2% to 4% [17-20]. The commonest sites of malignant change are the ilium (40%), scapula (11%) and pubic rami (11%) [21]. The scapula, clavicle and proximal humerus account for 18% of secondary chondrosarcomas in patients with HME [21]. Exostoses of the scapula have an increased probability of malignant change relative to all other exostoses (odds ratio 12) [4]. Prior to surgery, it is therefore important to ensure that the lesion is benign and if doubt exists the patient should undergo open biopsy and or excision.

## Conclusion

• Exostoses within the subacromial space causing impingement syndrome need to be ruled out in patients with HME

• Clinicians should have a low threshold to pursue further radiographic investigations, such as a MRI scan, of patients with HME presenting with subacromial impingement syndrome to exclude a symptomatic exostosis

• Arthroscopic excision of a benign subacromial exostosis is effective, offering a minimally invasive approach with relief of the patient’s symptoms

## Patient consent for publication

The patient described in this study gave her consent for her case to be described in this report and images to be used which have been anonymised.

## Competing interests

The authors, their immediate family, and any research foundation with which they are affiliated have not received any financial payments or other benefits from any commercial entity related to the subject of this article.

## Authors’ contributions

Both JMB and DEP were the Consultants in charge of the patient reported in this manuscript and were involved in the investigation and management. NDC composed the literature search and drafted the final manuscript. All authors read and approved the final manuscript.

## Pre-publication history

The pre-publication history for this paper can be accessed here:

http://www.biomedcentral.com/2052-1847/5/20/prepub
